# The Extracellular Matrix, the Silent ‘Architect’ of Glioma

**DOI:** 10.3390/biomedicines14010205

**Published:** 2026-01-17

**Authors:** Carmen Rubio, Javier Pérez-Villavicencio, Nadia F. Esteban-Román, Ángel Lee, Gervith Reyes-Soto, Moisés Rubio-Osornio

**Affiliations:** 1 Department of Neurophysiology, National Institute of Neurology and Neurosurgery, Mexico City 14269, Mexico; mrubio@innn.edu.mx; 2 Basic Sciences and Engineering Division, Department of Electrical Engineering, Iztapalapa Campus, Metropolitan Autonomous University, Mexico City 09340, Mexico; javierperezvillavicencio@gmail.com; 3 Biological Sciences and Health Division, Department of Biological Systems, Xochimilco Campus, Metropolitan Autonomous University, Mexico City 04960, Mexico; nadia.esteban.roman@gmail.com; 4 National Institute of Public Health, Cuernavaca 62100, Mexico; dr_angel_lee@yahoo.de; 5 Department of Surgery, National Institute of Cancer, Mexico City 14080, Mexico; drgervith@gmial.com; 6 Department of Neurochemistry, National Institute of Neurology and Neurosurgery, Av. Insurgentes Sur 3877, Mexico City 14269, Mexico

**Keywords:** extracellular matrix, glioma advancement, mechanotransduction, tumor diversity, ECM reorganization, therapeutic resistance

## Abstract

The brain’s extracellular matrix (ECM) serves as a dynamic and instructive regulator of glioma progression. The ECM provides structural support while integrating pharmacological and mechanical signals that influence glioma initiation, progression, and treatment resistance. Deviant ECM remodeling fosters tumor heterogeneity, invasion, and immune evasion by altering stiffness, composition, and cellular matrix signaling. We proposed that ECM remodeling in gliomas not only facilitates tumor growth and heterogeneity but also establishes advantageous biophysical and metabolic conditions that foster treatment resistance and recurrence. Our objective is to analyze current findings regarding the structural, biochemical, and mechanical roles of the brain ECM in glioma growth, emphasizing its contribution to tumor heterogeneity, mechanotransduction, immunological modulation, and its potential as a therapeutic target. Method: A comprehensive literature review was conducted using scientific databases including PubMed, Web of Science, and Scopus. Peer-reviewed literature published between 2000 and 2025 was selected for its relevance to ECM composition, stiffness, remodeling enzymes, extracellular vesicles, and mechanobiological processes in gliomas. Results: Recent investigations demonstrate that glioma cells actively alter the ECM by secreting collagens, laminins, and metalloproteinases, establishing a feedback loop that facilitates invasion and resistance. Discussion: Mechanical variables, such as ECM stiffness and solid stress, influence glioma growth, metabolism, and immune exclusion. Moreover, extracellular vesicles facilitate significant extracellular matrix remodeling and improve communication between tumors and stromal cells. The disruption of ependymal and subventricular extracellular matrix niches enhances invasion and cerebrospinal fluid-mediated signaling. The remodeling of the ECM influences glioma growth through interconnected biochemical, mechanical, and immunological mechanisms. Examining ECM stiffness, crosslinking enzymes, and vesicle-mediated signaling represents a potential therapeutic approach. Integrative methodologies that combine mechanobiology, imaging, and multiomics analysis could uncover ECM-related vulnerabilities to improve glioma treatment.

## 1. Introduction

The brain’s extracellular matrix (ECM) is increasingly acknowledged as a dynamic and instructive milieu that influences both normal neuronal function and pathological events, including gliomagenesis [1,2]. In a healthy brain, extracellular matrix components such as hyaluronic acid, proteoglycans, and glycoproteins facilitate neural plasticity, synaptic stabilization, and the maintenance of the blood–brain barrier (BBB) [3,4]. These chemicals are commonly expressed abnormally or change structurally in the glioma microenvironment, resulting in a permissive environment that encourages tumor invasion, metabolic flexibility, and immune evasion [5,6].

Gliomas, especially glioblastoma multiforme (GBM), exhibit great variation in their genetic and epigenetic profiles, as well as in their ECM composition and stiffness, which vary significantly across tumor locations [7,8]. The variations in ECM architecture influence cellular behavior via mechanotransduction pathways facilitated by integrins, focal adhesion kinases (FAKs), and downstream effectors such as YAP/TAZ and PI3K/AKT signaling [9,10]. Elevated ECM stiffness has been demonstrated to foster a mesenchymal-like phenotype, augmenting the motility and invasiveness of glioma cells [5,11]. Moreover, remodeling enzymes such as matrix metalloproteinases (MMPs) and lysis oxidases (LOXs) further affect the mechanical properties of the extracellular matrix (ECM) and regulate angiogenesis, establishing a feedback loop that sustains tumor progression [12,13].

The glioma ECM is characterized by a high concentration of brain-specific proteoglycans, including brevican, neurocan, and tenascin-C, which engage with cell surface receptors and affect intracellular signaling pathways [14,15]. These interactions can affect essential carcinogenic processes and contribute to therapeutic resistance. Tenascin-C has been associated with enhanced preservation of glioma stem cells (GSC) and increased resistance to radiation and temozolomide [16,17]. The extensive accumulation of hyaluronic acid in the glioma ECM stimulates CD44 and RHAMM receptors, facilitating tumor cell proliferation and migration via MAPK and ERK signaling [18,19]. These findings underscore the dual function of the ECM as both a structural and signaling element, capable of determining glioma cell fate and adaptability.

Despite these developments, many aspects of glioma ECM interactions remain poorly understood. A notable gap is the impact of prolonged biophysical stimulation on tumor development and cloning. Much contemporary research relies on short-term in vitro models or static analyses of patient biopsies, which fail to accurately reflect the temporal and geographical dynamics of extracellular matrix remodeling during tumor growth [20,21]. The role of non-tumor stromal cells, including astrocytes, pericytes, and tumor-associated macrophages, in the composition and rigidity of the ECM is still being investigated [22,23]. Comprehending these contributions is crucial, as stromal-tumor interactions can influence both invasion patterns and responses to immunotherapy and targeted therapies.

A significant problem is understanding how ECM composition affects medication transport and therapeutic effectiveness. The extensive, cross-linked ECM of the glomerular basement membrane can act as a physical impediment to the diffusion of chemotherapeutics and the infiltration of immune cells [24,25]. Moreover, integrin-mediated signaling and growth factors originating from the ECM can initiate survival pathways that mitigate the efficacy of conventional therapies, indicating that altering ECM cell interactions may enhance tumor sensitivity to therapy [26,27]. Recent strategies addressing ECM stiffness or composition, including LOX inhibition and the application of ECM-degrading nanoparticles, have demonstrated potential in preclinical investigations [28,29,30]. Translating these results into clinical success necessitates a comprehensive understanding of the temporal changes in the ECM and the variations in these modifications among genetic subtypes of glioblastoma.

Emerging ideas suggest that ECM remodeling may facilitate tumor growth and influence the immune microenvironment. Extracellular matrix molecules can affect the recruitment and activation of immune cells by altering cytokine gradients and receptor signaling [31,32]. Excessive accumulation of fibronectin and collagen I has been linked to an increased recruitment of tumor-associated macrophages and inhibition of T-cell function [33,34]. Consequently, ECM-targeted treatments may enhance immune infiltration while decreasing tumor invasiveness.

The ECM of the brain represents a dynamic and barely investigated element of glioma biology, incorporating mechanical, biochemical, and immunological signals. Enhancing our comprehension of glioma cell ECM remodeling and its effects on tumor heterogeneity and therapeutic response is essential for the advancement of next-generation treatment options. A comprehensive approach that combines in vivo imaging, biomaterials-based models, and multiomics profiling is essential for understanding these intricate connections [35,36]. Identifying and targeting ECM-driven vulnerabilities may provide new strategies to overcome the substantial resistance associated with malignant gliomas.

Understanding the content and organization of the extracellular matrix (ECM) in the glioma microenvironment and in the healthy brain is essential to elucidate how tumors progress. Proteoglycans and hyaluronan, which provide a soft, non-adhesive environment that inhibits cell migration, are commonly abundant in the healthy brain extracellular matrix. On the other hand, the abnormal deposition of fibrillar collagens and basement membrane components in the glioma extracellular matrix (ECM) results in increased stiffness and a pro-invasive environment (Table 1).

## 2. Extracellular Matrix Remodeling and Glioma

Recent findings indicate that glioblastoma (GBM) cells actively modify their extracellular matrix (ECM) environment through the secretion, degradation, and crosslinking of ECM components, rather than being passive occupants [1,83].

Glioma cells release several structural proteins, such as collagens, laminins, and fibronectin, which facilitate tumor growth and invasion [9,58]. Collagen type VI has been recognized as a crucial ECM component released by glioblastoma cells, modifying the biophysical characteristics of the tumor microenvironment and facilitating cell invasion via mechanotransduction pathways governed by integrins, including αvβ3, αvβ5, α6β1, and β1-containing integrins and β-catenin signaling. These integrins mediate cell–ECM adhesion, cytoskeletal rearrangement, and downstream activation of β-catenin–dependent transcriptional programs that promote migration and invasion in glioma cells [45,46]. This mechanism enhances the tumor’s capacity to traverse dense brain tissue and circumvent therapeutic measures [5]. The notion of mechanical memory, wherein glioma cells subjected to rigid ECM conditions preserve invasive characteristics when transitioning to more pliable settings, has lately garnered significant endorsement [84,85]. Cells cultivated on rigid surfaces display enduring alterations in cytoskeletal organization, YAP/TAZ activity, and chromatin remodeling that remain irrespective of the microenvironment, indicating epigenetic reinforcement of mechanical signals [86,87]. This mechanical priming promotes glioma recurrence and infiltration into surrounding brain tissue following surgical resection, highlighting the therapeutic significance of biomechanical adaptation [88,89]. Atomic force microscopy (AFM) and other nanoscale measuring techniques have revealed that ECM stiffness in glioblastomas is markedly variable, differing between the tumor core and invasive margin [5,37]. The rigid central regions are generally associated with high collagen crosslinking and increased levels of lysis oxidase (LOX), whereas the more flexible peripheral areas promote cellular mobility and tumor spread [28,90]. Regions exhibiting heightened stiffness are associated with recurrence locations and reduced survival rates, suggesting that biomechanical features serve as independent prognostic factors [38,91]. The findings underscore that glioma aggressiveness cannot be attributed exclusively to molecular genetics; it must also consider the mechanical context of the tumor microenvironment [22,92]. Extracellular vesicles (EVs), generated from gliomas, encompassing exosomes and macrovesicles, are crucial for ECM remodeling by conveying signaling chemicals, enzymes, and nucleic acids to adjacent stromal and glial cells [66,93]. These electric vehicles encompass MMPs, integrins, and microRNAs that regulate ECM deposition, angiogenesis, and immune cell activity [67,68]. For example, EVs containing MMP-9 and LOX-like enzymes promote matrix breakdown and stiffness, respectively, therefore enhancing tumor invasion [69,94]. Additionally, EVs activate astrocytes and microglia to produce ECM components such as fibronectin and periostin, which remodel the tumor border to promote glioma invasion [95,96]. This intercellular communication illustrates how glioma cells coordinate a collaborative restructuring of the ECM to facilitate development. Endothelial cells subjected to glioma-conditioned ECM display modified transcriptional patterns, characterized by the overexpression of ECM-associated genes such as COL27A1 and neuron, which can be recognized as possible prognostic indicators [97,98]. These alterations impact vascular integrity and facilitate the development of atypical tumor vasculature, marked by permeable, irregular arteries that intensify hypoxia and impede therapeutic efficacy [47]. Brain-specific ECM molecules, such as brevican and tenascin-C, further influence endothelial behavior by augmenting proliferation and facilitating pericyte recruitment, maintaining the abnormal vascular microenvironment [16,99].

Recent evidence indicates that ECM rigidity and composition influence metabolic plasticity in glioma cells [74,100]. Rigid surroundings induce a metabolic transition towards glycolysis and increased lactate synthesis, mechanisms that promote cellular migration and matrix disintegration via microenvironment acidification [75,101]. Simultaneously, ECM-mediated signaling via integrins and FAK activates downstream AKT/mTOR pathways, enhancing survival during hypoxic stress [9,26,102]. These findings associate mechanical and metabolic remodeling as synergistic processes propelling glioblastoma aggressiveness. From a therapeutic standpoint, focusing on ECM remodeling has emerged as an appealing but complex method [51,90,103]. LOX and MMP inhibitors have shown effectiveness in preclinical glioma models, decreasing stiffness and enhancing medication penetration [28,48]. Nonetheless, preliminary clinical trials have demonstrated restricted efficacy owing to compensatory activation of alternate remodeling pathways [26,104]. Innovative biomaterial-based strategies are currently being investigated to alter ECM characteristics, such as hydrogels that replicate brain stiffness for drug response analysis and nanoparticles engineered to locally dissolve ECM barriers and deliver therapeutic drugs [105]. A further interesting avenue of investigation entails the integration of ECM-targeting treatments with immunotherapy [78]. The thick and abnormal ECM in glioblastoma multiforme can serve as a physical and biochemical barrier to immunological infiltration, inhibiting T-cell function and promoting immune evasion [24,79]. Modifying ECM stiffness or composition can improve immune cell accessibility and augment the effectiveness of checkpoint inhibitors [38,79,80]. The ECM interacts with cytokine networks to influence macrophage polarization towards a tumor-promoting (M2-like) phenotype, a process that may be reversed through targeted ECM modification [81,106].

**Figure 1:**
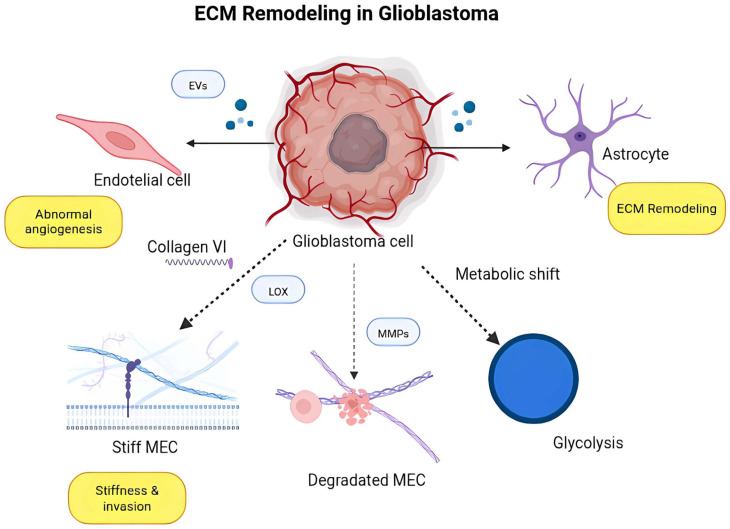
Glioblastoma cells drive pathogenesis through coordinated ECM remodeling. The tumor microenvironment is reprogrammed via three integrated mechanisms: (1) Structural ECM remodeling: GBM cells secrete and reorganize ECM components such as collagen VI, tenascin-C, fibronectin, and hyaluronan, while enzymes including lysyl oxidase (LOX) and matrix metalloproteinases (MMP-2 and MMP-9) mediate collagen cross-linking and ECM degradation. These alterations increase matrix stiffness and promote invasion through mechanotransduction pathways (e.g., FAK and YAP/TAZ activation). (2) Vascular dysregulation: GBM cells and stromal elements release pro-angiogenic factors such as VEGF-A, angiopoietin-2, and PDGF-BB, leading to structurally abnormal and leaky blood vessels that further modify the ECM. (3) Metabolic adaptation: GBM cells undergo a glycolytic shift driven by pathways involving HIF-1α, GLUT1, HK2, and LDHA, which feeds back to ECM remodeling through altered secretion and enzyme activity. Together, these ECM-mediated processes create a pro-tumoral microenvironment that sustains invasion, angiogenesis, and metabolic rewiring, positioning the ECM as a major therapeutic target in GBM. Created in BioRender. Villavicencio, X. (2026). https://BioRender.com/je9wvon, accessed on 13 January 2026.

In summary, recent advancements highlight that glioma growth is closely linked to ECM remodeling, which regulates cellular behavior via mechanical, biochemical, and metabolic signals [32,76,103]. Comprehending how glioma cells manipulate ECM dynamics provides a basis for discovering novel treatment targets that disrupt this interdependent interlinkage, as well as integrative methodologies that amalgamate omics studies, mechanobiology, and in vivo imaging, which are crucial for elucidating the intricacies of ECM glioma interactions [2,36,39,84]. The finding of ECM-derived biomarkers, including collagen VI, neurocan, and COL27A1, offers mechanistic insights as well as prospective diagnostic and prognostic tools for clinical use [8,14,15,45,97,98]. Ultimately, turning these findings into treatment solutions necessitates addressing the ECM not only as a structural component but also as a dynamic signaling system that perpetuates the malignant behavior of gliomas [1,5,12,13,14,17,19], (Figure 1).

## 3. Biophysical Stress Beyond Stiffness

Numerous research studies examining the tumor microenvironment have predominantly concentrated on the stiffness of the ECM, typically characterized by its elastic modulus. Nonetheless, another mechanical parameter, solid stress, characterized as the mechanical force applied by an expanding tumor mass that compresses adjacent tissues, has garnered relatively minimal focus [36,39,84,107]. In contrast to interstitial fluid pressure or matrix stiffness, solid stress constitutes a unique mechanical limitation that intensifies as the tumor proliferates inside restricted environments. This characteristic is particularly significant in gliomas due to the brain’s confinement within the inflexible skull, which restricts tissue expansion and intensifies compressive effects [39,84,108]. In various solid tumors, including breast and pancreatic cancer, solid stress has been demonstrated to attain sufficient magnitudes to collapse blood vessels, diminish perfusion, and induce hypoxia conditions that may facilitate tumor progression and treatment resistance [109,110]. Comprehending the manifestation of these physical forces inside the brain tumor microenvironment is crucial for elucidating glioma mechanopathology.

Recent technology advancements have facilitated the in vivo assessment of solid stress in brain tumors by high-resolution imaging and computer modeling. Researchers have shown that solid stress, assessed using magnetic resonance elastography and three-dimensional deformable registration, inversely correlates with patient survival in glioma, indicating it serves as a negative prognostic indicator [40,111]. This growing research redefines the traditional perspective of the tumor ECM; mechanical compression, rather than only stiffness, significantly impacts tumor physiology. Nevertheless, despite these encouraging advancements, the mechanisms by which solid stress influences ECM remodeling, matrix deposition, strain fields, and glioma cell behavior, including migration, proliferation, and mechanosensing, remain largely uninvestigated [11,36,37,39,52,112]. Additional research is required to clarify if solid stress is just a consequence of tumor growth or a significant factor in disease advancement.

From the standpoint of ECM dynamics, solid stress can initiate a series of mechanical and biological reactions that surpass mere matrix stiffening. Extended compression can stimulate tumor-associated fibroblasts or reactive astrocytes, enhancing the production of collagen, fibronectin, glycosaminoglycans, and other ECM components that modify tissue density and organization [31,36,41,113]. These structural modifications affect cell–matrix interactions via mechanotransduction pathways facilitated by integrins, focal adhesion kinase (FAK), Src/ERK, and YAP/TAZ signaling [9,10,114]. Thus, solid stress can establish a feedback loop in which the compressed ECM becomes denser and more anisotropic, hence exacerbating local mechanical stress. In the distinctive biomechanical environment of the brain marked by low stiffness and high water content, such compression may induce spatial confinement, alter ECM architecture, and modify strain gradients that influence glioma cell motility and invasiveness [11,42,115]. At the cellular level, solid stress directly impacts glioma behavior, affecting proliferation, motility, and mechanosensing. Experimental models of restricted tumor spheroids indicate that heightened compressive stress suppresses proliferation while enhancing an invasive phenotype, potentially via cytoskeletal remodeling and modified cell volume regulation [108,116,117]. In gliomas, areas subjected to significant compressive forces resulting from the enlarging tumor core or adjacent rigid ECM may promote cell migration instead of proliferation, thus facilitating the characteristic diffuse infiltrative pattern of these tumors [51,108,118]. Furthermore, solid stress can influence nutritional gradients and oxygen distribution by compressing microvessels, hence activating hypoxia-inducible signaling and metabolic reprogramming, which further exacerbates tumor aggressiveness [77]. Consequently, instead of merely being a passive consequence of tumor growth, solid stress may serve as an active modulator of glioma advancement and resistance to therapy.

**Figure 2:**
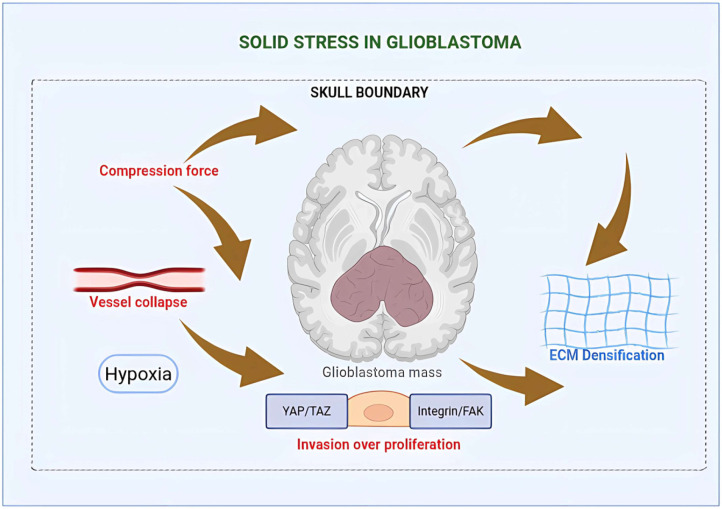
Solid stress in glioblastoma drives vascular collapse, hypoxia, and invasive phenotype switching. Schematic representation of solid stress-mediated mechanical forces in the glioblastoma (GBM) microenvironment and their multifaceted consequences on tumor progression. The growing tumor mass generates compressive forces (brown arrows) within the confined cranial space (dashed boundary), creating mechanical stress that propagates throughout the surrounding tissue. This mechanical compression induces vessel collapse, resulting in impaired blood perfusion and subsequent hypoxia. Concurrently, solid stress drives extracellular matrix (ECM) remodeling, characterized by increased matrix density and anisotropic fiber alignment (densified ECM mesh pattern). The integration of mechanical cues from both the stiffened ECM and the hypoxic microenvironment activates mechanotransduction pathways, including YAP/TAZ and integrin/FAK signaling cascades, which orchestrate a phenotypic switch in glioma cells. This mechanical reprogramming shifts cellular behavior from a proliferative to a highly invasive state, as illustrated by the elongated glioma cell with invadopodia-like protrusions migrating away from the tumor core. This biomechanical framework highlights how physical forces within the tumor microenvironment act as critical regulators of GBM malignancy, promoting therapeutic resistance and tumor dissemination through mechanically driven cellular plasticity. Created in BioRender. Villavicencio, X. (2026). https://BioRender.com/0f7phwc, accessed on 13 January 2026.

Recognizing the impact of solid stress on glioma biology offers new opportunities for clinical intervention from a translational and therapeutic standpoint. In vivo measurement of solid stress may serve as a novel imaging biomarker for patient stratification or predicting therapy response [111,119]. Furthermore, therapeutic modification of the ECM through enzymatic breakdown of collagen, inhibition of cross-linking enzymes, or pharmacological lowering of tissue tension may reduce compression, boost perfusion, and improve medication delivery to the tumor core [8,120]. In the restricted intracranial milieu, where minor volumetric alterations can yield significant repercussions, addressing solid stress may be crucial for enhancing the effectiveness of current therapies. However, the molecular connections among compressive stress, ECM remodeling, and glioma cell adaptability have yet to be thoroughly clarified. Future investigations that combine biophysical modelling, high-resolution imaging, and molecular profiling will be essential to understand how solid stress influences glioma development within the distinct biomechanical environment of the brain [43,51,118,119,121], (Figure 2).

## 4. Extracellular Matrix Remodeling in Non-Tumor Niches

The ependymal lining of the lateral ventricles, along with its associated ECM, establishes a specialized barrier between cerebrospinal fluid (CSF) and brain parenchyma. The ECM structures, referred to as fractions, play a significant role in influencing the local microenvironment [122,123,124]. Fractions are intricate, branched ECM structures situated beneath the ependymal cell layer that sequester soluble cerebrospinal fluid components and convey them to neural stem and progenitor cells located in the subventricular zone (SVZ) [62,125,126]. In glioblastoma (GBM), tumors located near the lateral ventricle exhibit increased aggressiveness and inferior clinical outcomes, indicating that the interplay among the tumor, cerebrospinal fluid (CSF), and subventricular zone (SVZ) niche enhances malignancy [127,128]. Recent results indicate that GBM compromises the integrity of the ependymal wall and modifies fraction shape inside the SVZ, potentially enabling the direct interaction of CSF-derived chemicals with the tumor microenvironment [127,129]. Examining these molecular interactions is essential for understanding how glioma-derived extracellular vesicles modify ECM structure and for developing strategies to prevent the formation of pro-invasive environments that precede tumor recurrence [31,130]. In an intracranial xenograft model, glioblastoma lesions adjacent to the lateral ventricle demonstrated tumor cell infiltration into the ependymal layer, along with a significant increase in the uptake of the fluorescent tracer DiI from the ventricular space into the tumor mass, signifying communication between cerebrospinal fluid and the tumor [127]. Furthermore, these periventricular tumors exhibited structural and functional anomalies in ependymal cells, characterized by diminished cilia length and density, lipid droplet accumulation, and downregulation of gap junction and channel proteins, including connexin-43 (Cx43) and aquaporin-4 (AQP4) [122,131]. These alterations indicate the deterioration of the ependymal barrier and the reorganization of the underlying ECM. Quantitative investigations indicated an increase in fraction density and a decrease in their average area, implying a dynamic remodeling response of the ventricular ECM to tumor invasion [122,132]. These observations prompt essential mechanistic inquiries: Which particular ECM components of fractions are modified during GBM invasion? The ECM in the subventricular zone (SVZ) niche consists of molecules including heparan sulphate proteoglycans (HSPGs), laminin-γ1, tenascin C, collagen IV, and various basement membrane glycoproteins that modulate the adhesion, migration, and proliferation of neural stem and progenitor cells [63,64,65]. Altering these components may yield an ECM composition that enhances the motility and invasiveness of glioma cells. Smaller, more abundant fractions may increase the overall surface area for binding heparin-affine growth factors from the CSF, thereby intensifying pro-tumoral signaling [31,49]. Furthermore, GBM cells produce several matrix-degrading enzymes, including matrix metalloproteinases (MMPs), which can modify or fracture ECM scaffolds, destabilizing the subventricular zone milieu and facilitating tumor cell dissemination along the ventricular wall [59].

From a therapeutic standpoint, focusing on ECM remodeling within the ependymal and SVZ niches may offer a unique approach to limit GBM invasion. If specific ECM molecules such as HSPGs, tenascin-C, or laminin-γ1 are upregulated or structurally altered to promote glioma migration and survival, they may act as molecular targets for inhibitory antibodies or small-molecule modulators aimed at restoring ECM integrity or obstructing tumor–niche interactions [49,56,120,133].

**Figure 3:**
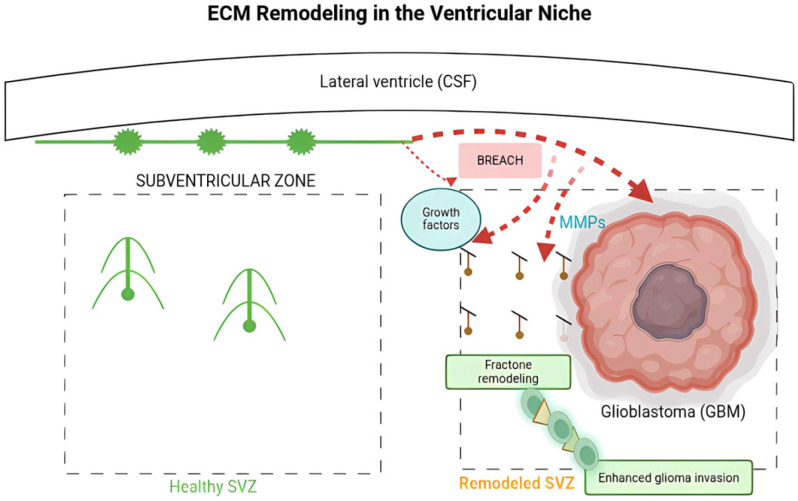
Glioblastoma co-opts the ventricular niche by reprogramming the SVZ ECM to create a pro-invasive corridor. Schematic depicting the pathological remodeling of the subventricular zone (SVZ) by a proximal glioblastoma (GBM) tumor. Tumor cells compromise the integrity of the ependymal barrier, enabling direct communication between the cerebrospinal fluid (CSF) and the tumor microenvironment. This breach facilitates the influx of CSF-derived trophic factors (e.g., FGF-2, BMP-4, BDNF) and the activity of tumor-secreted matrix metalloproteinases (MMP-2, MMP-9), which collectively drive the structural reorganization of specialized ECM structures called fractones. The transition from extensive, branched fractones to a dense network of smaller, fragmented structures enhances the surface area for pro-tumoral signaling (e.g., via CXCL12 gradients) and disrupts the native niche architecture. This remodeled, permissive ECM scaffold facilitates and promotes the diffuse invasion of glioma cells along the ventricular wall, demonstrating a novel mechanism for periventricular recurrence. Created in BioRender. Villavicencio, X. (2026). https://BioRender.com/opim6j1, accessed on 13 January 2026.

Furthermore, therapies designed to restore the ependymal barrier or inhibit CSF-tumor communication may diminish the availability of CSF-derived trophic substances that enhance malignancy. Notwithstanding these encouraging hypotheses, the fundamental molecular mechanisms are still elucidated, and it remains uncertain which fraction constituents experience the most substantial modifications, how these alterations affect the adhesive and signaling characteristics of the SVZ niche, or whether such changes can be reversed to mitigate tumor dissemination [123,125]. In conclusion, ECM remodeling in non-tumor niches, such as the ependymal zone and CSF interfaces, represents a vital yet underexplored facet of GBM biology. The disturbance of the ependymal lining, the structural reorganization of fractions, and the consequent interaction between the tumor and the cerebrospinal fluid milieu may provide a conducive scaffold that facilitates malignancy [70,128]. Subsequent investigations should focus on delineating the specific molecular modifications of the ECM in the periventricular areas, assessing their functional implications for glioma cell dynamics, and examining treatment approaches that either restore or counteract this pro-invasive ECM environment (Figure 3).

## 5. Extracellular Vesicles and Distant ECM Remodeling

**Figure 4:**
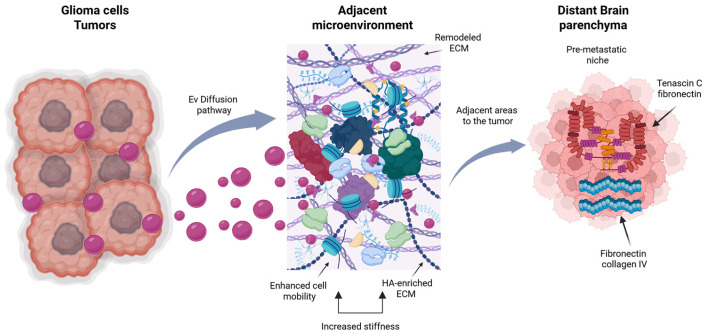
In this figure, glioma cells release EVs loaded with specific molecules such as podocalyxin and matrix metalloproteinases (MMPs). These EVs travel through the brain parenchyma and “educate” resident stromal cells (e.g., astrocytes). This education leads to a profound remodeling of the extracellular matrix (ECM) in adjacent areas, characterized by hyaluronic acid (HA) accumulation, increased deposition of tenascin C and fibronectin, and heightened tissue stiffness. These changes promote tumor cell motility and invasion. Furthermore, EV-mediated signals precondition distant sites, establishing a pro-invasive ECM rich in tenascin C and fibronectin, thereby creating pre-metastatic niches that facilitate tumor recurrence, highlighting ECM’s role as a “silent architect” of glioma progression. Created in BioRender. Esteban, N. (2026). https://BioRender.com/d8u018v, accessed on 13 January 2026.

Glioma cells secrete extracellular vesicles (EVs) that influence ECM deposition in adjacent cells, including astrocytes and glia [67,71]. This capacity to affect the adjacent microenvironment underscores a crucial communication channel between tumor and stromal cells that is frequently neglected [72,134]. Nonetheless, the overarching inquiry into the mechanisms of ECM remodeling in geographically far areas from the primary tumor mass is poorly comprehended [36,135]. The impact of glioma-derived EVs on ECM composition in areas distant from the tumor core is uncertain, potentially establishing “fields” for future invasion or recurrence [71,136]. Consequently, the diffusion lengths, temporal dynamics, and particular molecular cargo facilitating these processes must be clarified [137,138]. Recent data indicates that brain tumors, particularly glioblastoma (GBM), utilize extracellular vesicles (EVs) to “educate” cells within the tumor microenvironment, such as astrocytes, endothelial cells, and other glial populations, thereby facilitating ECM remodeling that enhances tumor invasion [36,94,134]. For example, extracellular vesicles (EVs) released by glioblastoma (GBM) cells can convey podocalyxin to astrocytes, prompting them to accumulate excessive hyaluronic acid (HA) and forming an HA enriched ECM that amplifies tumor cell motility and invasiveness [71,139]. The remodeled ECM not only delivers biochemical signals but also modifies the mechanical characteristics of the tumor microenvironment, enhancing stiffness and tissue tension that facilitate further glioma cell migration [51,53,84,140]. ECM remodeling involves breakdown, the production of new components, and matrix reorganization, processes closely associated with glioma progression and recurrence [31,60,141]. Most research, however, has concentrated on tumor-adjacent areas, with less investigation into the distance EVs can traverse or their role in the development of pre-metastatic niches in brain parenchyma remote from the original tumor [70,72,142]. Recurrent gliomas exhibit a denser, more rigid ECM that is enriched in molecules such as tenascin C, fibronectin, and collagen IV, which promote invasion and contribute to therapeutic resistance [31,57]. These findings suggest that glioma cells can alter the microenvironment much beyond their initial site via biochemical gradients or extracellular vesicle diffusion [67,68,69,94,135,137]. Comprehending the diffusion lengths and dynamics of extracellular vesicle transport in cerebral tissue presents a significant difficulty. Recent biophysical studies demonstrate that extracellular vesicles (EVs) can navigate dense or restricted matrices; however, their mobility is markedly affected by tissue hydration, ECM porosity, and local stiffness [73,137]. In the brain, where the ECM is notably dense and compositionally distinct, these physical restrictions may restrict the range of extracellular vesicles or selectively direct their pathways of distribution [51,71,72,135,143]. The temporal aspect is similarly significant: What is the duration required for ECM alterations generated by EVs to manifest as a functioning pro-tumoral niche that facilitates migration or recurrence? Currently, there is no definitive in vivo data that delineates these timelines [60,94]. The precise molecular components involved in ECM remodeling are still being actively researched. ECM formed from gliomas has been demonstrated to transport matrix metalloproteinases (MMP-2, MMP-9), CD44, and integrins, which can degrade ECM components, enhance adhesion, and activate migratory signaling in recipient cells [31,50,51,54,144]. These cargo molecules may allow EVs to function as “mobile microenvironmental modulators,” creating conducive settings for tumor progression even at remote locations [72,82,94,145]. Examining these molecular interactions is essential for understanding how glioma-derived extracellular vesicles modify ECM architecture and for developing strategies to prevent the formation of pro-invasive environments that lead to tumor recurrence [31,146], (Figure 4).

## 6. Spatial Heterogeneity of ECM Components

Bulk assays have revealed significant differences in ECM gene and protein expression across glioma tissues; however, single-cell and spatially resolved studies of ECM components within the tumor microenvironment remain in the preliminary phase [51,147]. Novel high-resolution tools, including spatial proteomics, imaging mass cytometry, and single-cell RNA sequencing, now facilitate the characterization of ECM heterogeneity at micron-scale resolution [148,149]. These methodologies enable researchers to investigate the variation in ECM composition in regions proximal to tumor cells versus more distant stromal areas, uncovering gradients of certain ECM components, including collagens, laminins, fibronectin, and proteoglycans [51,97,150]. Spatial profiling aids in elucidating the evolution of ECM stiffness, crosslinking, and biochemical signaling throughout glioma growth, hence affecting pathways associated with migration, integrin activation, and mechanotransduction [151,152]. This ECM variability is closely associated with invasive behavior, facilitating perivascular infiltration and therapeutic resistance through matrix-mediated signaling via receptors such as CD44, ITGB1, and DDR1 [153,154]. Moreover, geographic and single-cell transcriptome analyses reveal that glioma cells actively alter their microenvironment by secreting ECM-modifying enzymes, including MMP2, MMP9, and LOX, which facilitate tissue remodeling and invasion [51,55]. Integrating these single-cell and geographical ECM maps may yield essential insights into the molecular interactions between tumor cells and their extracellular environment, uncovering novel treatment targets that impede ECM-mediated invasion and recurrence [151,155]. Recent studies employing multiplexed ion beam imaging (MIBI) and co-detection by indexing (CODEX) have revealed that ECM organization differs markedly between infiltrative zones and necrotic cores, with specific collagen isoforms and proteoglycans enriched at invasive fronts, where they colocalize with mesenchymal-like glioma cells [156,157]. Furthermore, spatial transcriptomics platforms such as Visium and GeoMx have enabled the mapping of ECM gene expression patterns across entire tumor sections, revealing regional variations in ECM composition that correlate with molecular subtypes and treatment responses [158,159]. The spatial heterogeneity of the ECM is inherently connected to the mechanical properties of its microenvironment. Areas with elevated ECM density, frequently abundant in cross-linked collagens and tenascin-C, demonstrate enhanced stiffness, subsequently activating mechanotransduction pathways like YAP/TAZ and FAK in glioma cells. This localized mechanical signaling promotes the acquisition of a highly invasive, mesenchymal-like phenotype, especially near the tumor-brain interface, where stiffness gradients are most pronounced [44,144].

This spatial non-uniformity is orchestrated by extracellular vesicles (EVs). Glioma-derived EVs carry a targeted assortment of matrix-modifying enzymes (e.g., matrix metalloproteinases, lysyl oxidase) and signaling molecules (e.g., microRNAs, growth factors) to designated regions, thereby “pre-conditioning” the adjacent parenchyma. The communication mediated by extracellular vesicles results in localized regions with modified extracellular matrix composition and stiffness, thereby promoting invasion in a non-random, spatially defined manner, frequently along white matter tracts or perivascular spaces [160,161].

**Figure 5:**
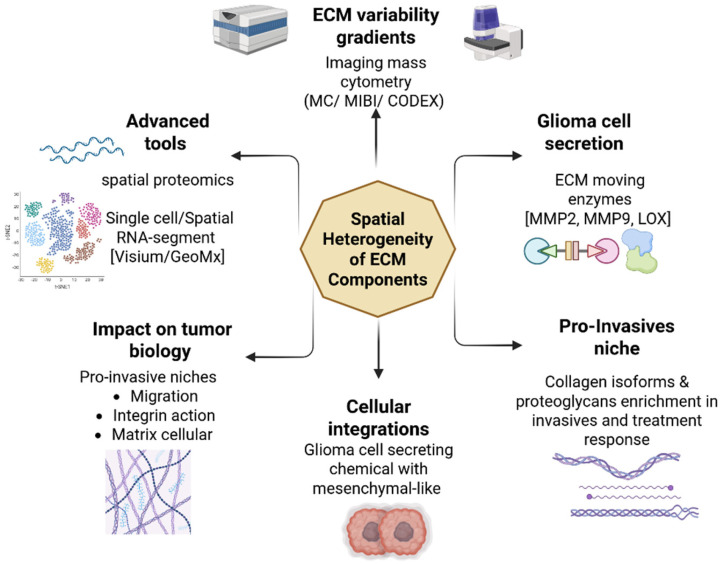
Analysis of ECM Heterogeneity in Glioma Using High-Resolution Spatial Platforms. The diagram underscores the necessity of moving beyond conventional bulk assays to achieve a comprehensive understanding of the Extracellular Matrix (ECM). The deployment of advanced technologies, including spatial transcriptomics (GeoMx, Visium), imaging mass cytometry (CODEX), and spatial proteomics, facilitates the precise mapping of regional variations in ECM composition (specific collagens, laminins) and mechanical properties (stiffness). Critically, this spatially resolved information is essential for correlating glioma’s molecular subtypes with ECM gene expression patterns and therapeutic response. Created in BioRender. Esteban, N. (2026). https://BioRender.com/irkmr7v, accessed on 13 January 2026.

The ECM in non-tumor niches also demonstrates significant heterogeneity. The sub-ventricular zone (SVZ) and the perivascular niche, where ECM components such as hyaluronan and tenascin-C are abundant, function as favorable pathways for glioma cell migration. The modification of ECM in non-tumoral regions, frequently induced by reactive astrocytes and microglia, disrupts normal tissue architecture, fostering an environment conducive to invasion and perhaps aiding in the development of distant recurrence sites [162,163]. Comprehending the specific ECM composition in these niches is crucial for the development of targeted therapies aimed at obstructing these invasion pathways (Figure 5).

## 7. Extracellular Matrix-Based Biomaterials and Extracellular Matrix Hydrogels

Preliminary investigations have commenced to examine the therapeutic potential of hydrogels produced from ECM of non-neoplastic tissues, such as urinary bladder ECM, which have demonstrated the ability to diminish glioma cell viability in vitro and postpone recurrence in experimental models [30,44]. Investigations suggest that decellularized ECM hydrogels can provide bioactive signals that affect the glioma microenvironment, potentially reducing tumor recurrence after surgical resection [144,160]. These hydrogels mimic the components of the natural extracellular matrix, preserving critical proteins, glycosaminoglycans, and growth factors that influence cellular behavior and differentiation [161,162]. In glioma research, ECM hydrogels are considered a novel method for altering the tumor microenvironment and inhibiting the proliferation of residual tumor cells [44,163]. Nonetheless, the application of these ECM-based biomaterials to in vivo brain glioma models is a significant difficulty owing to the distinctive biochemical and biomechanical properties of the brain [144,164]. The brain ECM markedly contrasts with peripheral tissues due to its high content of hyaluronic acid, proteoglycans, and glycoproteins, while exhibiting a scarcity of fibrillar collagens, which are prevalent in sources like the urinary bladder or small intestinal submucosa [165,166]. Consequently, biomaterials sourced from non-neural ECM may not accurately replicate the native composition or mechanical compliance of the central nervous system [161,167].

Improving the ECM source, purification techniques, and crosslinking chemistry are crucial for achieving compatibility with brain tissue [168,169]. The optimal administration method, whether through injection, implantation, or in situ gelation, has not yet been standardized for the sensitive and restricted cerebral environment [137,164]. A notable concern is the interaction of these hydrogels with the host’s extracellular matrix following surgery [144,170]. Following glioma resection, the brain undergoes inflammatory and wound-healing processes that alter extracellular matrix composition, leading to a reactive and pro-fibrotic microenvironment [31,171]. The introduction of a biomaterial in this context may either reduce or exacerbate such reactions, depending on its degradation properties and immunomodulatory characteristics [168,172]. Optimal ECM scaffolds should offer transient structural support and anti-tumoral bioactivity while progressively dissolving without inducing inflammation [161,173]. Excessive rigidity or durability of the scaffold may hinder glial scar formation, disrupt neuronal connections, or provoke chronic neuroinflammation [174,175]. Thus, achieving a balance between mechanical integrity and biological safety is a crucial design aspect for ECM hydrogels in neurological applications [30,165]. Furthermore, real translational obstacles persist regarding their utilization in the post-surgical cavity [31,176]. Injectable ECM hydrogels must occupy irregular voids without elevating intracranial pressure or mass effect [137,164]. They must also withstand early destruction by matrix metalloproteinases while permitting regulated remodeling by host cells [13,61,174]. It is equally crucial to guarantee that the biomaterial does not unintentionally offer adhesion signals or growth factors that facilitate glioma cell survival or migration [144,177].

**Figure 6:**
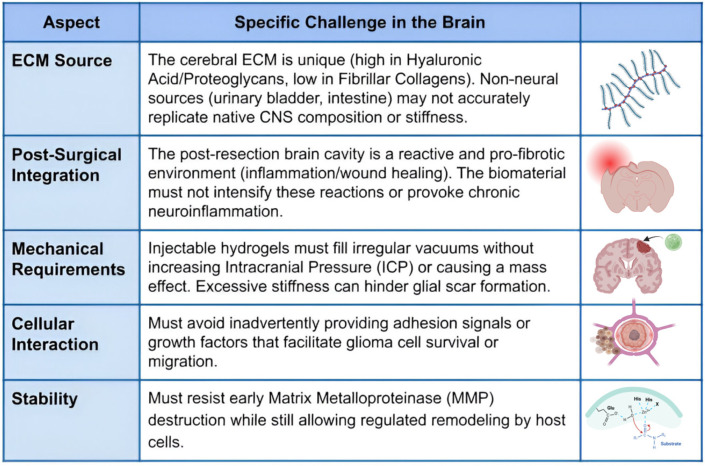
Decellularized ECM hydrogels function as scaffolds that release bioactive signals (proteins, glycosaminoglycans, growth factors) to suppress the proliferation of residual tumor cells. The overarching goal is to reduce glioma cell viability in vitro and postpone recurrence in vivo. These materials must provide transient structural support and anti-tumoral bioactivity while progressively dissolving without causing neuroinflammation. Future directions involve utilizing brain-derived ECM (e.g., porcine) to achieve more suitable biochemical and mechanical properties and improve integration, as well as creating hybrid hydrogels that combine brain-specific ECM components with synthetic polymers to attain tunable mechanical properties and controlled drug release. Created in BioRender. Esteban, N. (2026) https://BioRender.com/5hggem7, accessed on 13 January 2026.

Preclinical investigations utilizing decellularized brain ECM have demonstrated promising outcomes for neurological restoration post-stroke; however, analogous assessments regarding glioma recurrence prevention are limited [166,168]. Brain-derived ECM scaffolds may offer more appropriate biochemical cues and mechanical properties compared to non-neural sources, potentially improving integration and reducing adverse reactions [176,178]. Recent studies have explored the use of porcine brain ECM hydrogels that maintain the native glycosaminoglycan composition and growth factor profiles, demonstrating enhanced neuronal survival and reduced glial scarring in rodent models [30,167]. Thus, creating ECM scaffolds that proficiently suppress glioma proliferation while preserving the physiological capabilities of normal brain ECM presents a substantial challenge in biomaterials-focused neuro-oncology [165,179]. Future directions include engineering hybrid hydrogels that combine brain-specific ECM components with synthetic polymers to achieve tunable mechanical properties and controlled drug release [176,180]. Additionally, functionalization of ECM scaffolds with anti-tumor agents, such as temozolomide-loaded nanoparticles or immune checkpoint inhibitors, may enhance their therapeutic efficacy while maintaining their biocompatibility [161,181], (Figure 6).

## 8. Discussion

The alteration of the brain ECM by glioma cells is a crucial factor in tumor growth, treatment resistance, and recurrence. The ECM serves not just as a structural scaffold but also as a dynamic signaling platform that integrates mechanical, biochemical, and metabolic signals to influence glioma behavior. The information presented indicates that glioma aggressiveness is influenced not only by genetic or epigenetic modifications but also by the reciprocal interactions between tumor cells and their mechanical microenvironment. The anomalous composition of the extracellular matrix (ECM), altered stiffness, and dysregulated remodeling enzymes, such as matrix metalloproteinases (MMPs) and lysyl oxidases (LOXs), collectively establish a favorable and varied environment that facilitates glioma cell plasticity and survival.

Recent discoveries indicate that ECM stiffness and solid stress are separate but interrelated biophysical forces that influence glioma mechanopathology. Stiffness influences mechanotransduction pathways via integrins, FAK, and YAP/TAZ signaling, while solid stress from tumor growth in the restricted cranial cavity generates compressive pressures that modify perfusion, nutrition gradients, and cell migration patterns. These pressures jointly induce hypoxia, metabolic reprogramming, and an invasive phenotype, ultimately enhancing the characteristic diffusivity of glioblastoma. The mechanical characteristics of the glioma microenvironment not only mirror tumor growth but also actively influence it. Alongside these mechanical effects, biochemical alterations of the ECM by glioma-derived extracellular vesicles (EVs) extend the tumor’s influence beyond its immediate confines. The transmission of matrix remodeling enzymes, proteoglycans, and adhesion molecules via extracellular vesicles facilitates ECM reorganization in remote brain regions, possibly establishing pre-invasive habitats that promote recurrence. This process highlights the systemic aspect of glioma ECM interactions, wherein spatially distant remodeling events can predispose brain tissue to further invasion. In this setting, the identification of EV cargoes such as MMPs, CD44, and integrins emerges as a viable path for diagnostic and therapeutic investigation. Another overlooked area pertains to ECM remodeling in non-tumor niches, especially at the ependymal zone and cerebrospinal fluid (CSF) interfaces. Gliomas located adjacent to the lateral ventricles have increased invasiveness and an unfavorable prognosis, probably because of the compromise of the ependymal barrier and structural modifications in specialized EMC structures of the subventricular zone (SVZ). These alterations promote molecular interchange between cerebrospinal fluid and tumor, potentially supplying glioma cells with a reservoir of growth factors and cytokines that enhance malignancy. The interaction among the ventricular EMC, neural stem cell niches, and infiltrating tumor cells may constitute a pivotal axis in glioma propagation that necessitates further examination. Targeting ECM remodeling therapeutically offers substantial potential as well as considerable challenges. The potential exists in disrupting the tumor’s physical and biochemical support system, which may improve the effectiveness of standard treatments. Strategies aimed at inhibiting ECM crosslinking (e.g., LOX inhibitors), degrading excess matrix deposition (e.g., matrix metalloproteinase inhibitors), or modifying mechanical stress have shown significant preclinical efficacy, particularly in improving drug delivery and immune cell infiltration [164,165].

The clinical translation of these approaches is significantly hindered by the inherent challenges presented by the glioma microenvironment. The challenges encompass:

1. Tumor adaptability and biomechanical compensation: Gliomas demonstrate significant plasticity. Focusing on a specific ECM component or remodeling enzyme frequently results in the compensatory activation of alternative signaling pathways or the upregulation of additional matrix components, thereby circumventing the therapeutic intervention and contributing to treatment resistance [166,167].
2. Inadequate specificity in ECM-targeting: The extracellular matrix in the brain is essential for normal neural function. Broad-spectrum ECM-targeting agents exhibit insufficient specificity to differentiate between the pathological tumor matrix and the healthy brain matrix, resulting in significant neurotoxicity and restricting clinical applicability [168].
3. Tumor heterogeneity, characterized by the spatial and temporal variability of the extracellular matrix (ECM), suggests that a uniform therapeutic approach is improbable to yield efficacy across the entire tumor mass or throughout the disease progression. The invasive margin, distinguished by elevated stiffness and particular extracellular matrix components, necessitates an alternative approach compared to the less rigid tumor core [169].

## 9. Clinical Trials and Limitations

We incorporate a broader examination of clinical trials aimed at ECM-related mechanisms in glioblastoma. Despite the encouraging results of numerous preclinical studies, most clinical trials have not succeeded in improving the survival rates of GBM patients. Trials involving broad-spectrum MMP inhibitors, such as Marimastat, demonstrated limited efficacy primarily due to a lack of specificity and systemic toxicity [170]. Recent strategies, including those aimed at integrins (e.g., cilengitide, an inhibitor of αvβ3 and αvβ5 integrins), initially demonstrated potential but ultimately did not achieve primary endpoints in Phase III trials, underscoring the difficulties in converting preclinical results into clinical advantages [171]. The limitations of current approaches arise from the aforementioned issues, particularly the tumor’s capacity for compensatory signaling and the challenge of attaining adequate ECM targeting specificity without systemic side effects. The future of glioma treatment likely entails the integration of molecular and mechanical targeting to simultaneously disrupt the tumor’s physical and biochemical support systems. The integration of ECM-targeted therapies with immunotherapy or anti-angiogenic treatments may yield synergistic benefits, particularly given the ECM’s role as a physical barrier to immune cells and its involvement in fostering vascular irregularities [172,173]. Innovative approaches, including nanomedicine-based delivery systems aimed at locally degrading the extracellular matrix (ECM) or targeting specific ECM-receptor interactions (e.g., CD44-hyaluronan), are crucial for addressing existing limitations and attaining a more balanced and clinically relevant perspective.

## 10. Conclusions and Perspectives

The alteration of the brain ECM by glioma cells is a crucial factor in tumor growth, treatment resistance, and recurrence. The ECM serves not just as a structural scaffold but also as a dynamic signaling platform that integrates mechanical, biochemical, and metabolic signals to influence glioma behavior. The information presented herein highlights that glioma aggressiveness is not exclusively determined by genetic or epigenetic modifications but arises from the reciprocal interaction between tumoral cells and their mechanical microenvironment. Anomalous ECM composition, modified stiffness, and dysregulated remodeling enzymes, including MMPs and LOXs, collectively create a conducive and diverse environment that supports glioma cell plasticity and survival.

Recent discoveries indicate that ECM stiffness and solid stress are separate but interrelated biophysical forces that influence glioma mechanopathology. Stiffness influences mechanotransduction pathways via integrins, FAK, and YAP/TAZ signaling, while solid stress from tumor growth in the restricted cranial cavity generates compressive pressures that modify perfusion, nutrition gradients, and cell migration patterns. These pressures jointly induce hypoxia, metabolic reprogramming, and an invasive phenotype, ultimately enhancing the characteristic diffusivity of glioblastoma. The mechanical characteristics of the glioma microenvironment not only mirror tumor growth but also actively influence it. Alongside these mechanical effects, biochemical alterations of the ECM by glioma-derived extracellular vesicles (EVs) extend the tumor’s influence beyond its immediate confines. The transfer of matrix remodeling enzymes, proteoglycans, and adhesion molecules through extracellular vesicles aids in ECM reorganization in distant brain regions, potentially creating pre-invasive environments that encourage recurrence. This process underscores the systemic nature of glioma extracellular matrix interactions, where spatially remote remodeling events may predispose brain tissue to additional invasion. The identification of EV cargoes, including MMPs, CD44, and integrins, represents a promising avenue for diagnostic and therapeutic research. Another neglected aspect involves ECM remodeling in non-tumor niches, particularly at the ependymal zone and cerebrospinal fluid (CSF) interfaces. Gliomas situated near the lateral ventricles exhibit heightened invasiveness and a poor prognosis, likely due to the disruption of the ependymal barrier and structural alterations in specialized extracellular matrix components of the subventricular zone (SVZ). These alterations promote molecular interchange between cerebrospinal fluid and tumor, potentially supplying glioma cells with a reservoir of growth factors and cytokines that enhance malignancy. The interaction among the ventricular EMC, neural stem cell niches, and infiltrating tumor cells may constitute a pivotal axis in glioma propagation that necessitates further examination. Targeting extracellular matrix remodeling presents both an opportunity and a challenge in therapeutic contexts. Strategies aimed at inhibiting ECM crosslinking, such as LOX inhibitors, degrading excess matrix deposition, or modifying mechanical stress, have shown preclinical effectiveness in improving drug delivery and immune cell infiltration. Clinical translation has been hindered by compensatory signaling and a limited understanding of extracellular matrix changes over time. The integration of ECM-targeted therapies with immunotherapy or anti-angiogenic treatments may yield synergistic benefits, particularly in light of the ECM’s role in immune suppression and vascular abnormalities. The future of glioma treatment is expected to involve the integration of molecular and mechanical targeting to simultaneously disrupt the tumor’s physical and biochemical support systems.

## Figures and Tables

**Table 1:** Key differences between samples of healthy brain tissue and glioma (Extracellular Matrix’s (ECM) components, structural features, and functional implications).

Functional Implication in Tumor Progression	Glioma/Tumor Microenvironment ECM	Normal Brain Tissue ECM	Component/Characteristic	References
Elevated stiffness promotes tumor cell migration, invasion, proliferation, and resistance to apoptosis.	High (Increased deposition of collagen and fibronectin)	Low (Soft tissue, dominated by proteoglycans)	Stiffness	[5,11,37,38,39,40,41,42,43,44]
Provides fibrillar scaffolds for cell invasion and facilitates tumor angiogenesis.	Increased Collagen I, III, V, and VI (in tumor stroma and perivascular space)	Predominantly Collagen IV (in vascular basement membrane); low abundance in the parenchyma.	Collagens	[8,12,45,46,47,48,49,50,51]
Increases interstitial pressure, promotes cell motility and invasion through interaction with receptors like CD44.	Overexpression and fragmentation; presence of low molecular weight HA.	Mainly high molecular weight, uniform and regulated distribution.	Hyaluronan (HA)	[18,49,52,53,54,55]
Anti-adhesive molecule that promotes cell migration. Induces chemotherapy resistance (via PI3K/Akt) [16] and is a target for immunotherapy (CAR-T) [56].	Marked overexpression, especially at the invasive front and in hypoxic areas	Very low or absent expression in the adult brain, except in neurogenic niches.	Tenascin C (TnC)	[7,14,35,48,49,51,57]
Mediates cell adhesion, migration, and contributes to chemotherapy resistance.	High expression in the perivascular stroma and tumor microenvironment.	Low expression, mainly associated with vasculature.	Fibronectin (FN)	[1,7,14,27,33,48,51]
ECM degradation, facilitating invasion, angiogenesis, and the release of sequestered growth factors.	High activity (e.g., MMP-2, MMP-9, MMP-14)	Low activity, strictly regulated for physiological turnover.	Matrix Metalloproteinases (MMPs)	[13,48,50,58,59,60,61]
It is hypothesized that glioma stem cells (GSCs) exploit this ECM and factor rich niche for maintenance and to promote recurrence.	Glioblastoma infiltrates and alters the SVZ, potentially co-opting fractones.	Specialized ECM structures in the subventricular zone (SVZ), rich in HSPGs (Perlecan), that sequester growth factors (FGF2).	Fractones (SVZ Niche)	[62,63,64,65]
“Educate” stromal cells (astrocytes, microglia) to deposit a pro-tumoral ECM (HA, TnC), prepare pre-metastatic niches, and suppress immunity.	Tumor EVs loaded with oncoproteins, miRNAs, MMPs (e.g., PODXL, MMP-9), and pro-remodeling factors.	Physiological EVs for cell-to-cell communication.	Glioma-derived Extracellular Vesicles (EVs)	[66,67,68,69,70,71,72,73]
Acidosis and hypoxia induce the expression of ECM remodeling genes (COL, LOX, TnC) and promote an invasive and immunosuppressive phenotype.	Metabolic acidosis (lactate accumulation from aerobic glycolysis). Hypoxia.	Oxidative metabolism, neutral pH.	Microenvironment Metabolism	[74,75,76,77]
Cross-link collagen fibers, increasing ECM stiffness and stability, which promotes invasion and enhances integrin signaling.	Overexpression of LOX and LOXL family members.	Low basal expression.	Modifying Enzymes (LOX/LOXL)	[28,29,30,45]
Creates a profoundly immunosuppressive microenvironment (“cold tumor”) that is a major barrier to immunotherapy.	Remodeled and immunomodulatory ECM. Increased stiffness and components like HA and TnC suppress T-cell infiltration and function, while recruiting and polarizing macrophages (GAMs) towards a pro-tumoral phenotype.	ECM supports neuronal function and homeostatic immune surveillance.	Immune Cell Interaction	[78,79,80,81,82]

## Data Availability

No new data were created or analyzed in this study. Data sharing is not applicable to this article.

## Abbreviations

ECM	Extracellular Matrix
GBM	Glioblastoma Multiforme
BBB	Blood–Brain Barrier
MMPs	Matrix Metalloproteinases
LOXs	Lysyl Oxidases
GSC	Glioma Stem Cells
FAKs	Focal Adhesion Kinases
YAP/TAZ	Yes-Associated Protein/Transcriptional Coactivator with PDZ-binding motif
PI3K/AKT	Phosphoinositide 3-Kinase/Protein Kinase B
MAPK	Mitogen-Activated Protein Kinase
ERK	Extracellular signal-Regulated Kinase
AFM	Atomic Force Microscopy
Evs	Extracellular Vesicles
CSF	Cerebrospinal Fluid
SVZ	Subventricular Zone
Cx43	Connexin-43
AQP4	Aquaporin-4
HSPGs	Heparan Sulphate Proteoglycans
HA	Hyaluronic Acid
DiI	1,1′-dioctadecyl-3,3,3′,3′-tetramethylindocarbocyanine (DiIC18(3))
MRE	Magnetic Resonance Elastography
MIBI	Multiplexed Ion Beam Imaging
CODEX	Co-Detection by Indexing
